# Dysregulation of Sirtuin 2 (SIRT2) and histone H3K18 acetylation pathways associates with adverse prostate cancer outcomes

**DOI:** 10.1186/s12885-017-3853-9

**Published:** 2017-12-20

**Authors:** Shivashankar Damodaran, Nathan Damaschke, Joseph Gawdzik, Bing Yang, Cedric Shi, Glenn O. Allen, Wei Huang, John Denu, David Jarrard

**Affiliations:** 1 Department of Urology, School of Medicine and Public Health, University of Wisconsin, Madison, WI 53705 USA; 2 Carbone Comprehensive Cancer Center, University of Wisconsin, Madison, WI 53705 USA; 3 Department of Pathology and Laboratory Medicine, University of Wisconsin School of Medicine and Public Health, Madison, WI 53705 USA; 4 Department of Biomolecular Chemistry, University of Wisconsin, Madison, WI 53706 USA; 5 Wisconsin Institute for Discovery and the Morgridge Institute for Research, University of Wisconsin, Madison, WI 53715 USA; 6 Molecular and Environmental Toxicology Program, University of Wisconsin, Madison, WI 53706 USA; 7 John P. Livesey Chair in Urologic Oncology, Associate Director Translational Research, Carbone Cancer Center, University of Wisconsin School of Medicine and Public Health, 7037 WIMR, 1111, Highland, Avenue Madison WI 53705 USA

**Keywords:** Epigenetic modifications in PCa, Histone H3 acetylation, Cancer progression, SIRT2 loss

## Abstract

**Background:**

Histones undergo extensive post-translational modifications and this epigenetic regulation plays an important role in modulating transcriptional programs capable of driving cancer progression. Acetylation of histone H3K18, associated with gene activation, is enhanced by P300 and opposed by the deacetylase Sirtuin2 (SIRT2). As these enzymes represent an important target for cancer therapy, we sought to determine whether the underlying genes are altered during prostate cancer (PCa) progression.

**Methods:**

Tissue microarrays generated from 71 radical prostatectomy patients were initially immunostained for H3K18Ac, P300 and SIRT2. Protein levels were quantified using VECTRA automation and correlated with clinicopathologic parameters. The Cancer Genome Atlas (TGCA, *n* = 499) and Gene Expression Omnibus (*n* = 504) databases were queried for expression, genomic and clinical data. Statistics were performed using SPSSv23.

**Results:**

Nuclear histone H3K18Ac staining increases in primary cancer (*p* = 0.05) and further in metastases (*p* < 0.01) compared to benign on tissue arrays. P300 protein expression increases in cancer (*p* = 0.04) and metastases (*p* < 0.001). A progressive decrease in nuclear SIRT2 staining occurs comparing benign to cancer or metastases (*p* = 0.04 and *p* = 0.03 respectively). Decreased SIRT2 correlates with higher grade cancer (*p* = 0.02). Time to Prostate Specific Antigen (PSA) recurrence is shorter in patients exhibiting high compared to low H3K18Ac expression (350 vs. 1542 days respectively, *P* = 0.03). In GEO, SIRT2 mRNA levels are lower in primary and metastatic tumors (*p* = 0.01 and 0.001, respectively). TGCA analysis demonstrates SIRT2 deletion in 6% and increasing clinical stage, positive margins and lower PSA recurrence-free survival in patients with SIRT2 loss/deletion (*p* = 0.01, 0.04 and 0.04  respectively). In this dataset, a correlation between decreasing SIRT2 and increasing P300 mRNA expression occurs in tumor samples (*R* = −0.46).

**Conclusions:**

In multiple datasets, decreases in SIRT2 expression portend worse clinicopathologic outcomes.

Alterations in SIRT2-H3K18Ac suggest altered P300 activity and identify a subset of tumors that could benefit from histone deacetylation inhibition.

**Electronic supplementary material:**

The online version of this article (10.1186/s12885-017-3853-9) contains supplementary material, which is available to authorized users.

## Background

Prostate cancer (PCa) is the most common cancer by incidence and second most lethal cancer in American males [1]. DNA methylation and histone tail modification are two key epigenetic processes that play vital roles in prostate cancer progression [2, 3]. Histone post-translational modifications (PTMs) including acetylation, methylation, and phosphorylation exist in a highly specific fashion and function to influence gene transcription by interconverting chromatin from its permissive and repressive states [4]. Histone acetyl transferases (HAT) and deacetylases (HDAC) are enzymes that add or remove acetyl groups on the lysine residues in the N terminal chains of histones [5]. Addition of acetyl groups to histones affects the stability of nucleosomes and alters the access of DNA binding proteins to their recognition sites. The presence of acetylated histones in nucleosomes near transcriptional start sites are generally associated with gene transcription. The histone modulating enzymes (HATs and HDACs, respectively) can be targeted to specific regions of the genome and show degrees of substrate specificity, properties that are consistent with a role in maintaining a dynamic, acetylation-based epigenetic code [6].

Altered abundance of acetyl marks on lysine residues on histone 4 (H4) and Histone 3 (H3) are common in several cancers and have prognostic information [7, 8] Furthermore, it is possible to differentiate between benign and malignant prostatic tissue based on certain epigenetic characteristics [9]. For example, Seligson et al. have shown stratifying primary prostatectomy tissue samples by overall acetylated H3 and H4 immunostaining predicted tumor recurrence in patients with low-grade prostate cancers [10]. Similarly, acetylation of H3 and trimethylation of H4 have also been used in combination with preoperative serum PSA to predict the likelihood of recurrence in high-grade prostate cancers [11].

P300 protein is a ubiquitously expressed HAT that regulates a diverse range of cellular functions including cell growth, differentiation and survival [12, 13]. While P300 is capable of acetylating several histone lysines in vitro, H3K18 appears to be a target unique to P300 as P300 knockdown causes global hypoacetylation at this site [14, 15]. The catalytic activity of P300 is regulated by an evolutionary conserved autoacetylation loop at K1499 and this loop is modified by several factors including deacetylases such as the Sirtuins [16, 17]. Sirtuins are a group of proteins that function in cellular metabolism, chromatin stability, and DNA repair [18]. Among the seven known mammalian Sirtuins, SIRT2 can function as a tumor suppressor and its knockdown has been shown to induce gender-specific tumorigenesis in mice [19]. SIRT2 is a key deacetylase of the P300 K1499 site in vitro and in vivo studies and SIRT2 knockdown causes P300 to attain a hyperacetylated state, which is associated with higher acetyltransferase activity [20]. The deacetylase activity of SIRT2 maintains H3K18Ac in check, thereby exerting a controlling influence on the transcription process. P300 on the other hand can potentiate its own activity by autoacetylation and by inhibiting the activity of SIRT2 by acetylation. Thus, there is reciprocal regulation between P300 and SIRT2 that serves to maintain epigenetic homeostasis.

We recently demonstrated using a novel histone array chip that P300 acetylation activity is markedly upregulated during the development of castration-resistant prostate cancer (CRPC).^25^ In the present study, we questioned whether histone H3K18Ac acetylation, and its regulating genes P300 and SIRT2, are altered in a subset of early tumors and whether this correlates with clinicopathologic outcomes.

## Methods

### Tissue microarray

A previously described Tissue Microarray (TMA) [20] constructed from 71 radical prostatectomy specimens included 23 benign prostatic hyperplasia (BPH), 25 High Grade Prostatic Intraepithelial Neoplasia (HGPIN), 71 Prostate cancer (PCa) and 47 cancer-associated benign and 22 metastases. In sum, 388 cores (duplicates) were utilized. Patients involved in the study provided written informed consent and the study was approved by our Institutional Review Board. The mean follow-up duration was 13.6 years. Clinicopathological data is provided in Table 1. Cancer and HGPIN cores were chosen in such a way to not include >10% intervening normal glands.

**Table 1:** Clinicopathological correlation with H3K18Ac, P300 and SIRT2 expression analyzed by immunohistochemistry

Clinical		H3K18Ac	(p)	SIRT2	(p)	P300	(p)
Extraprostatic Extension	No (46)	0.371(0.316–0.434)		0.0152(0.010–0.019)		0.135(0.125–0.150)	
	Yes (24)	0.375(0.308–0.414)	0.71	0.0132(0.011–0.017)	0.17	0.145(0.125–0.162)	0.09
Seminal Vesicle Invasion	No (54)	0.373(0.318–0.431)		0.015(0.011–0.019)		0.135(0.125–0.15)	
	Yes (17)	0.365(0.306–0.414)	0.41	0.011(0.010–0.016)	0.12	0.146(0.128–0.165)	*0.03*
Laterality	U/L (4)	0.380(0.323–0.410)		0.010(0.0085–0.013)		0.125(0.118–0.136)	
	B/L (67)	0.371(0.31–0.422)	0.92	0.015(0.011–0.018)	0.16	0.137(0.125–0.153)	0.20
Margins	No (44)	0.371(0.312–0.429)		0.0157(0.011–0.019)		0.139(0.126–0.158)	
	Yes (26)	0.373(0.306–0.414)	0.79	0.012(0.010–0.017)	0.07	0.132(0.125–0.147)	0.46
Clinical stage	II (42)	0.371(0.316–0.439)		0.015(0.010–0.019)		0.134(0.125–0.150)	
	III (12)	0.358(0.308–0.401)		0.013(0.010–0.017)		0.137(0.126–0.153)	
	IV (16)	0.396(0.311–0.414)	0.64	0.016(0.010–0.018)	0.58	0.146(0.126–0.163	0.13
PSA level (ng/ml)	0–5 (6)	0.343(0.296–0.456)		0.011(0.009–0.015)		0.136(0.124–0.146)	
	5–10 (35)	0.372(0.313–0.414)		0.015(0.012–0.018)		0.133(0.124–0.152)	
	>10 (12)	0.365(0.307–0.425)	0.96	0.011(0.010–0.019)	0.15	0.145(0.134–0.160)	0.51
Gleason score							
(3 + 3, 3 + 4)	Low/Int (35)	0.371(0.321–0.441)		0.015(0.012–0.019)		0.128(0.121–0.142)	
**(**4 + 3,4 + 4, 4 + 5)	High (19)	0.345(0.298–0.412)	0.15	0.011(0.009–0.015)	*0.02*	0.134(0.124–0.152)	0.68

Relative staining intensity of H3K18Ac, SIRT2 and P300 (mean, standard deviation expressed in 0D units) compared to clinicopathological correlates

### Staining

Slide preparation was done as previously described for VECTRA analysis. The TMA slides were taken through routine deparaffinization and rehydration, pretreated with endogenous peroxidase block and retrieval buffer. Slides were then rinsed with dH2O, Tris Buffered Saline (TBS), and then TBS with Tween (TBST), followed by protein blocking at room temperature. E cadherin antibodies were used for epithelial compartmentalization. Multiple antigen labelling was done with 3, 3′ – diaminobenzidine (DAB) for staining of H3K18Ac and P300, while peroxidase chromogen VIP® (V-VIP; Vector Labs) was used for counter staining.

For image analysis and quantification of the staining intensity, VECTRA system was used. Cores with <5% epithelial component or loss of tissue were excluded from the analysis. Nuance system and inform 1.2™ software (Caliper Life Sciences, Hopkinton, MA) were used to for building spectral libraries on the basis of target signals of the three stained parameters. This system allows automated quantitation of fluorescent staining on a per-cell basis and selection of cellular subsets (nucleus versus cytoplasmic) for analysis of target signals.

### Database analyses

The Cancer Genome Atlas (TCGA) prostate adenocarcinoma samples were queried using cBioPortal for Cancer Genomics (www.cbioportal.org). All 499 prostate adenocarcinoma samples were analyzed for SIRT2 and p300 using RNA-sequencing and copy number alterations. Clinical data for all samples was downloaded using the TCGA bio links (Bioconductor) package in R. Samples were grouped based on SIRT2 copy number status (SIRT2 deletion versus SIRT2 diploid) and compared to clinical and pathologic variables. Gene Expression Omnibus (GEO) was queried for data set GSE 6919 (Chandran et al.) [21] using SIRT2 probes. There were 504 samples from 168 patients which also included 52 samples from 17 organ donors with prostates free of any pathological abnormalities. RNA expression levels were compared between benign, primary and metastatic PCa.

### Statistical analysis

Staining patterns of H3K18Ac, P300 and SIRT2 were individually compared between benign, BPH, HGPIN, cancer and metastatic tissues by using student’s t test. For each of the three subcategories, nuclear, cytoplasmic and total cellular staining pattern was also quantified to act as internal control and to improve accuracy. However, for meaningful prediction of activity, only the nuclear staining pattern was taken into account. Clinicopathological correlates analyzed were extraprostatic extension, seminal vesicular invasion, positive surgical margins, laterality, clinical stage, PSA levels and recurrence. For each of the above parameters, a student T test or ANOVA was used for significance calculation. Kaplan Meyer curves compared PSA recurrence free survival between two groups based on median H3K18Ac nuclear staining pattern.

PCa data from the TCGA database was divided into 2 cohorts based on the deletion of SIRT2 gene. SIRT2 data was available for 492 out of 499 (98.5%) and patients with amplified SIRT2 10(2%) were excluded from analysis. Cohort 1 included patients with partial or deep deletion of SIRT2 gene and cohort 2 included patients with an intact diploid set of SIRT2 gene. Clinical and pathological data were compared between the SIRT2 deleted and SIRT2 intact group using Student’s T Testing or ANOVA. Kaplan Meyer curves were constructed for biochemical (PSA) recurrence-free survival between the 2 groups. Statistical analysis was done with SPSS v 23 (IBM, Armonk, New York). All tests were two-tailed and a *P* value <0.05 was considered statistically significant.

## Results

### H3K18Ac immunostaining increases in primary and metastatic PCa

**Fig. 1:**
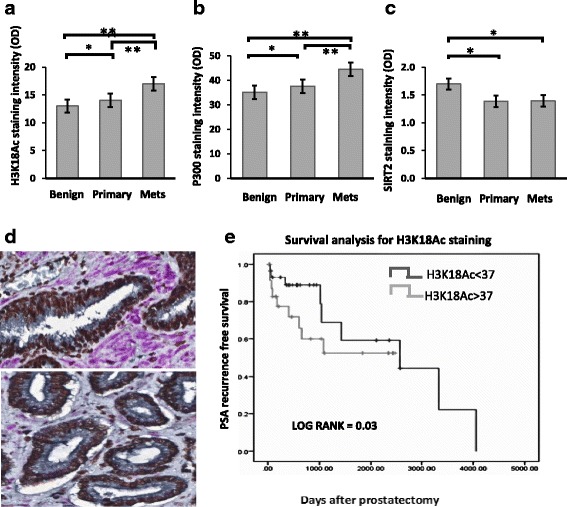
Concurrent immunostaining demonstrates H3K18Ac and P300 increase and SIRT2 decreases during prostate cancer progression. Mean nuclear staining intensity of H3K18Ac, P300 and SIRT2 compared between benign, primary and metastatic PCa. Immunohistochemistry was quantitated using VECTRA and inform software as described. **a** Nuclear levels of H3K18Ac, (**b**) p300 and (**c**) SIRT2 levels in benign, primary and metastatic PCa. (*p* < 0.05 indicated by * and <0.01 indicated by **). **d**. Staining patterns in representative benign (top) and malignant (bottom) tissues. In this triple stained image, H3K18Ac stains brown (Chromo DAB) and SIRT2 stains purple (Vector VIP), while E-cadherin used for compartmentalization stains black. (**e**). Increased H3K18Ac acetylation identifies patients at increased risk of PCa recurrence. Kaplan Meyer curves comparing PSA recurrence-free survival between high and low H3K18Ac staining. H3K18Ac staining on a per-core basis was measured using the Vectra platform in all tissues. Patients were stratified into high or low H3K18Ac levels based on median core expression. There was a statistically significant PSA-free survival advantage in the low H3K18Ac staining group. (1542 vs 350 days, *p* = 0.03)

Total histone acetylation levels at H3K18 are largely determined by the histone acetyl transferases P300 and opposed by the deacetylase SIRT2. Utilizing immunohistochemistry and VECTRA automated intensity analysis, nuclear staining patterns were determined for H3K18Ac, P300 and SIRT2 on tissue arrays from 71 primary PCa specimens of which 42 were organ confined (pT2) and 29 higher stage (pT3 and 4). Patient characteristics (Additional file 1: Table S1) included a median PSA of 6.8 (5.4–9.9) with 15 (33%), 20 (55%) and 19 (13%) of patients having low, intermediate and high Gleason Scores. H3K18Ac nuclear staining increases in primary (*p* = 0.05) and metastatic tissues (*p* = 0.007) compared to normal associated benign tissues, with increases also noted between primary and metastatic tissues (*p* = 0.03) (Fig. 1A; 1D).

### Total P300 increases and SIRT2 immunostaining decreases in primary and metastatic PCa

Since P300 protein is a key acetylator of H3K18Ac, we analyzed the expression pattern of this protein in the prostate TMAs. Total nuclear P300 protein staining increases when benign tissue is compared to primary and metastatic cancer tissues (*p* = 0.04 and <0.001, respectively) with metastatic tissue greater than primary (*p* = <0.001) (Fig. 1B). We hypothesized the staining pattern of SIRT2, an inhibitor of P300, would decrease given the increased hyperacetylation of H3K18Ac seen in cancer. In tissues, nuclear SIRT2 staining shows a decline from benign to malignant and metastasis (*p* = 0.04 and 0.03 respectively), but not between cancer and metastatic groups (*p* = 0.18) (Fig. 1C). Thus, an increase in nuclear P300 and decrease in SIRT2 was demonstrated during progression from benign to primary and metastatic cancer.

### Decreased SIRT2 and increased P300 correlate with clinicopathological parameters and increased H3K18Ac with biochemical recurrence

Clinical and pathological data for the 71 primary patients used for TMA construction were then compared to protein levels of SIRT2, P300 and H3K18Ac (Table 1). Greater P300 correlates with seminal vesicle invasion, increased Gleason Sum (4 + 3, 4 + 4, 4 + 5) demonstrates decreased SIRT2 (*P* = 0.02) compared to lower Gleason scores cancers. There was a trend towards lower SIRT2 expression and higher P300 expression with other worse pathological outcomes although not statistically significant. Kaplan Meyer curves for 10-year PSA recurrence-free survival constructed based on median H3K18Ac staining levels for the cancer group show worsened outcomes for the group below the median cut off value (350 vs. 1542 days, *p* = 0.03) (Fig. 1E).

### SIRT2 deletion in large genomic analysis datasets demonstrate worse prognosis

**Fig. 2:**
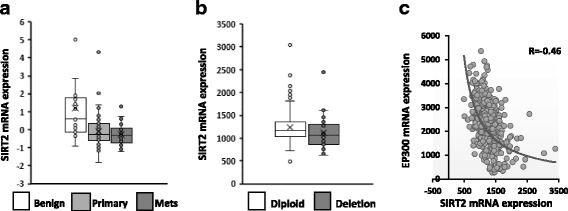
**a** mRNA expression analysis from human genome data in Genome Expression Analysis (GEO) data repository showing differential expression of SIRT2 mRNA between benign and primary PCa (*p* < 0.01) and between benign and metastasis (*p* = 0.004). **b** SIRT2 copy number alterations from Genomic Identification of Significant Targets in Cancer (GISTIC), showing the median and interquartile range of mRNA expression for SIRT2 in prostate cancer. There were 30 patients with SIRT2 deletions in the TCGA data base, including both shallow (*n* = 27) and deep (*n* = 3), which were compared to the 452 patients with diploid SIRT2. The mRNA expression levels were greater in the diploid compared to the deletion group. (*p* value = 0.03). **c** EP300 m RNA (gene for P300) expression shows a negative correlation with the mRNA expression of SIRT2. (Courtesy – TCGA database)

To extend this analysis, we then queried the Gene Expression Omnibus (GEO) for SIRT2 expression data based on results provided by Chandran et al. (GSE 6919)^21^. SIRT2 mRNA expression levels are significantly lower in primary tumor (*p* = 0.01) and metastasis (*p* = 0.004), compared to benign prostatic tissues (Fig. 2A). P300 expression and clincal correlates are not available in this dataset.

To further investigate this pathway, genomic profiles from 499 prostate adenocarcinoma cases indexed in the TCGA databases were retrieved. Currently no protein data is available, however based on our findings of decreased SIRT2 we queried the database for SIRT2 copy number alterations and compared clinical and pathological correlates between patients with decreased SIRT2 copy number (shallow and deep deletions) and patients with no copy number alterations of SIRT2 (diploid). There are 30 (6%) patients with SIRT2 gene deletions and 452 (90.5%) patients were diploid with respect to SIRT2. Amplification is rare (2%). The median mRNA expression level is lower in SIRT2 deleted patients compared to the SIRT2 intact group (*p* = 0.03) (Fig. 2B). Clinical data is available for this dataset and we observed higher clinical stage, greater chance of margin positive status at surgery and a higher likelihood of clinical recurrence in the subset of PCa patients with SIRT2 deletions (Table 2). Kaplan Meyer analysis trends towards shorter PSA recurrence-free survival for the SIRT2 deleted subgroup compared to patients with diploid SIRT2 (2406 vs 3550 days; *p* = 0.17) (not shown). The mRNA expression profiles for SIRT2 and P300 were also directly compared and show an inverse correlation (*R* = −0.46; Fig. 2C).

**Table 2:** SIRT2 deletion versus clinicopathological outcomes in the prostate cancer TCGA database (*n* = 499)

	SIRT2 Deleted (*n* = 30)	SIRT2 Diploid (*n* = 452)	P
Age (Median/IQR)	64(61.75–66.75)	61(56–66)	
TNM stage			
T1	0	2(0.4%)	
T2	5(16.67%)	164(40.2%)	
T3	22(73.34%)	276(61.2%)	
T4	3(10%)	9(2%)	*0.01* ^a^
N1	8(27%)	79(17.4%)	0.22
M+		3	
Recurrence			
Yes	10(33.34%)	81(17.9%)	
No	15(50%)	290(64%)	*0.04* ^a^
Bilateral	29(96%)	435(96%)	1
LND^a^ dissection	29(96%)	421(90%)	0.7
Nodes (Median/IQR)	7(4–16)	9(5–16)	
Positive margins	16(53%)	152(33.6%)	*0.04* ^a^
Complete remission	9 (30%)	164(33.6%)	0.8
Partial remission	4 (13%)	29(6.4%)	0.14
Biochemical relapse	3 (20%)	48(10.6%)	0.13

^a^Clinicopathological outcomes were compared between patients with diploid and deleted SIRT2. Patients with SIRT2 deletion had higher clinical ‘T’ stage, increased chance of recurrence and greater odds of positive surgical margins

## Discussion

Nucleosome compaction is an important component in the aberrant epigenetic transcriptional repression seen in cancer. Compacted nucleosomes block gene expression by sequestering the nucleotide sequences necessary for transcription factor binding and RNA polymerase recruitment [22]. Histone acetyl transferases (HATs) and histone deacetylases (HDACs) dictate the presence of acetyl marks [23, 24], which regulate the openness of chromatin. Thus, the processes for adding and removing these marks have become putative targets for cancer therapy. Their abundance during prostate cancer remains understudied. In this study using roughly 1100 patients across multiple databases, we find that the HAT P300 and its target H3K18Ac increase during prostate cancer development and progression, while the HDAC SIRT2 decreases. Increased H3K18Ac and decreased SIRT2 serve as biomarkers for worse clinical outcomes after radical prostatectomy.

Site-specific acetylation due to a deficiency of HDACs has been shown to be one of the epigenetic determinants of PCa progression [24]. We had previously identified using a novel screen for altered histone modifying enzymes that H3K18Ac increases in an androgen-independent PCa cell line compared to its androgen dependent isogenic counterpart [25]. H3K18Ac levels marked increases in acetylation activity in that study. In the current study, we observed that the nuclear levels of H3K18Ac increase when comparing benign to primary and metastatic PCa. Furthermore, a stratified analysis based on the median H3K18Ac staining intensity showed that higher levels of H3K18Ac are an independent predictor of PSA recurrence-free survival irrespective of the grade of the tumor. This increased risk of recurrence with H3K18Ac was suggested in an earlier prostate cancer dataset [26]. Our results showing predictive value across all grades expands and contrasts with the findings of Seligson et al. who found that H3K18Ac and H3K4me2 levels could be used in combination for predicting the risk of recurrence in low Gleason (<7) prostate cancer [10]. The reason for the contrasting findings could be due to the methodology, as western blotting and semi-quantitative staining measurements were previously used, whereas the current study employs state of the art automated quantitative immunofluorescence (VECTRA) image analysis [27].

H3K18Ac hyperacetylation involves loss of P300 acetyltransferase inhibition in part by a reduction of SIRT2 deacetylase activity [20, 28]. P300 catalytic function is tightly regulated in cells through several mechanisms despite its broad substrate specificity. First, autoacetylation leading to activation occurs only in gene promoters during transcription initiation. Secondly, SIRT2 inhibits P300 acetyltransferase activity by removing activating acetyl groups from the P300 catalytic domain [29]. This is counterbalanced by acetylation of SIRT2 by P300 to retain its enzymatic activity [30]. Notably we were not able to assess phosphorylated P300 in this study due to lack of a specific antibody.

Loss of function of SIRT2 releases the inhibitory control on P300, leading to progressive hyperacetylation of H3K18Ac [31]. We confirmed loss of SIRT2 function occurs as an important event in the hyperacetylation sequence in a subset of tumors by comparing protein as well as mRNA expression levels between benign and PCa tissues from multiple databases. Significantly lower levels of SIRT2 in tumor tissues were expressed compared to normal. SIRT2 deletion also has prognostic value as TCGA genomic data with clinicopathological data correlates with increased likelihood of recurrence, higher clinical stage and a lower likelihood of negative margins.

SIRT2 has been shown to have a tumor suppressor function and its deletion has been linked to tumorigenesis in murine models and certain tumors [18, 32]. Our extensive analysis, including automated intensity scoring, suggests SIRT2 loss correlates with aggressive cancer and occurs early in prostate tumorigenesis. Previous work has demonstrated SIRT2 knockdown results in an accumulation of acetylated P300 in vivo without an increase in the level of total P300 [20]. However, our analysis of 500 TCGA prostate samples revealed a decrease in the levels of SIRT2 mRNA expression correlates strongly with increasing P300 transcript levels. This raises the possibility of a negative feedback control of P300 expression by SIRT2 that requires further exploration.

Limitations of the present study are its retrospective nature, lack of standardized biomarker analysis across databases and incomplete clinical data for some patients within these large databases restricting the recurrence-free survival calculations. In addition, the expression of these genes does not represent enzyme activity. However as noted in our previous work, HK18Ac correlates with increased acetylation activity in prostate cancer cell lines [24, 25]. The present work highlights the need for further prospective studies to assess the predictive accuracy of the SIRT2-H3K18Ac signature.

## Conclusion

Dysregulation of the P300-Sirt2-H3K18Ac pathway occurs during the development and progression of  prostate cancer. Alterations in SIRT2 as well as H3K18Ac predict adverse outcomes. SIRT2 loss and H3K18Ac gain appear to reflect the hyperacetylation mediated by P300 and their determination by genomic testing could help identify that subset of patients likely to benefit from HAT inhibitor therapy.

## Additional file

Additional file 1: Table S1. Clinicopathological characteristics of TMA array patients This table describes the clinical and pathological characteristics of the 71 patients, whose samples were used for TMA construction. (DOCX 13 kb)

## Abbreviations

ANOVA: Analysis of Variance
CRPC: Castrate Resistant Prostate Cancer
DAB: 3,3′ – diaminobenzidine
GEO: Gene Expression Omnibus
H3 and H4: Histone 3 and 4
H3k18Ac: Acetylated Histone H3k18
HAT: Histone Acetyl Transferase
HDAC: Histone Deacetylase
mRNA: Messenger RNA
PCa: Prostate cancer
PSA: Prostate Specific Antigen
PTM: Post Translational Modification
RNA: Ribonucleic Acid
SIRT2: Sirtuin 2
TBS: Tris Buffered Saline
TBST: TBS with Tween
TCGA: The Cancer Genome Atlas
TMA: Tissue Micro Array
V-VIP: Peroxidase chromogen VIP®
