# Supplementary material for: Dysregulation of Sirtuin 2 (SIRT2) and histone H3K18 acetylation pathways associates with adverse prostate cancer outcomes
Source: BMC Cancer. 2017 Dec 20;17:874. doi: 10.1186/s12885-017-3853-9 (PMC5738829; doi:10.1186/s12885-017-3853-9)
Supplement: Additional file 1: Table S1. — Clinicopathological characteristics of TMA array patients This table describes the clinical and pathological characteristics of the 71 patients, whose samples were used for TMA construction. (DOCX 13 kb) [file 12885_2017_3853_MOESM1_ESM.docx]

**Additional file 1: Table S1. Clinicopathological characteristics of TMA array patients.**

| Variable |  | median (IQR) |
| --- | --- | --- |
| Age |  | 60 (55 – 65) |
| PSA |  | 6.8 (5.4 – 9.9) |
| Tumor Volume (cc) | | 19%(7 – 36%) |
| [Gleason score*](https://www.ncbi.nlm.nih.gov/pmc/articles/PMC4398352/table/pone.0124366.t001/#t001fn001) | |  |
| 6 |  | 15 (21.1%) |
| 7 |  | 32 (45%) |
| 8 |  | 13 (18.3%) |
| 9 |  | 11 (15.4%) |
| Stage |  |  |
| II |  | 42 (59.1%) |
| III |  | 12 (16.9%) |
| IV |  | 17 (23.9%) |
| Lymph node |  |  |
| No |  | 54 (76%) |
| Yes |  | 17 (23.9%) |
| Extraprostatic Extension | |  |
| No |  | 46 (64.7%) |
| Yes |  | 24 (33.8%) |
| SV Involvement | |  |
| No |  | 54 (76%) |
| Yes |  | 17 (23.9%) |
| [Biochemical Recurrence**](https://www.ncbi.nlm.nih.gov/pmc/articles/PMC4398352/table/pone.0124366.t001/#t001fn002) | |  |
| No |  | 32 (45%) |
| Yes |  | 23 (32.3%) |
